# Decision-making considerations for single-dose HPV vaccination, including drivers of schedule adoption or switch: insights from immunisation stakeholders in 19 low-income and middle-income countries

**DOI:** 10.1136/bmjgh-2024-018779

**Published:** 2026-02-16

**Authors:** Erica N. Rosser, Ishani Sheth, Megan D. Wysong, Sunny Roy, Casey Geddes, Rupali J. Limaye, Joseph G. Rosen

**Affiliations:** 1 Department of International Health, Johns Hopkins University Bloomberg School of Public Health, Baltimore, Maryland, USA; 2 Department of Health, Behavior, and Society, Johns Hopkins University Bloomberg School of Public Health, Baltimore, Maryland, USA

**Keywords:** Decision Making, Vaccines, Qualitative study, Public Health, Global Health

## Abstract

**Introduction:**

Emerging evidence of durable immunogenicity from mono-dose human papillomavirus vaccination (HPVV) prompted the WHO to recommend a single-dose HPVV schedule in December 2022. There is, however, limited understanding of processes and considerations influencing country adoption of the updated HPVV dosing schedule recommendation.

**Methods:**

We identified four archetypes characterising countries’ progress along the HPVV introduction and single-dose adoption continua. From September 2023 to February 2024, we purposefully sampled and conducted semistructured interviews with immunisation stakeholders representing Ministries of Health, Gavi-funded technical assistance partners, civil society organisations and multilateral agencies from African and Asian low-income and middle-income countries. Using multicycle, iterative thematic analysis, we identified factors enabling the adoption of the HPVV single-dose recommendation, as well as constraints to rendering a decision on the HPVV dosing schedule.

**Results:**

We interviewed 66 stakeholders across 19 countries with mature HPVV programmes (n=11) or forthcoming national HPVV introductions (n=8), as well as countries adopting (n=10) or undecided about (n=9) the single-dose schedule. Stakeholders conveyed enthusiasm for single-dose HPVV, citing the following anticipated benefits: higher HPVV schedule completion and coverage, especially in underimmunised populations; costs saved from operational reconfigurations and reduced vaccine procurement demands, particularly for countries transitioning out of Gavi co-financing in a vaccine supply-constrained environment; and optimised vaccine stock management capacity, importantly for countries pursuing new vaccine introductions for multiple antigens simultaneously. Factors demotivating HPVV single-dose schedule adoption or delaying decision-making included: limited localised evidence of long-term immunologic protection from single-dose HPVV; off-label product use liabilities; costs/resources required for retraining the health workforce in countries with mature HPVV programmes; and potential for widening HPVV coverage inequities, notably in countries with elevated HIV burdens.

**Conclusions:**

Coupled with the WHO’s endorsement, the perceived benefits of single-dose HPVV consistently outweighed the anticipated risks, even when these risks delayed country-level HPVV schedule-related decision-making.

**WHAT IS ALREADY KNOWN ON THIS TOPIC**

- Human papillomavirus vaccination (HPVV) is the cornerstone of global cervical cancer elimination efforts, but given the novelty of evidence indicating mono-dose HPVV’s non-inferiority to a two-dose regimen, there is limited understanding of how low-income and middle-income countries (LMICs) are navigating HPVV schedule-related decision-making in the presence of updated global guidelines endorsing single-dose HPVV.

**WHAT THIS STUDY ADDS**

- LMIC decisions to adopt or switch to a single-dose HPVV schedule reflect a dynamic interplay between scientific evidence, programme costs and (dis)advantages, and the potential for significant public health impact. Robust scientific evidence and endorsements from global scientific authorities (ie, WHO) provided the impetus for LMICs to proceed with adopting or switching to an HPVV single-dose schedule, even amidst off-label use concerns.

**HOW THIS STUDY MIGHT AFFECT RESEARCH, PRACTICE OR POLICY**

- While the anticipated benefits of single-dose HPVV tended to outweigh its perceived risks, it is imperative that countries mobilise functional governance structures to render timely, evidence-informed and autonomous decisions related to the HPVV dosing schedule.

## Introduction

 Cervical cancer is one of the most common cancers among cisgender women worldwide, ranking fourth in both incidence (660 000 new cases) and mortality (350 000 deaths) in 2022.^1^ Cervical cancer also disproportionately impacts women and their families in low-income and middle-income countries (LMICs), where 94% of cervical cancer deaths worldwide occur.^1^ Cervical cancer burdens in LMICs are largely driven by low human papillomavirus vaccination (HPVV) coverage, suboptimal implementation of cervical screening and treatment services, and sociostructural forces (eg, vaccine hesitancy/misinformation, constraints to health-related decision-making autonomy by girls and women, limited vaccine availability or access to vaccination services).^1^

In 2020, at the World Health Assembly, 194 countries made the historic decision to work together to eliminate cervical cancer and avert 5 million deaths by 2050.^2^ Vaccination is the cornerstone of the WHO’s global elimination strategy, together with screening and treatment. Since 2012, 16.3 million girls have been protected against the leading cause of cervical cancer through HPVV.^3^ There continues to be great progress, with more than 32 countries receiving full support from Gavi to introduce HPVV into their national immunisation programmes, resulting in millions of girls receiving HPVV through Gavi co-financing.^3^

In December 2022, based on emerging evidence of substantial immunogenicity and high vaccine efficacy from studies of mono-dose HPVV, the WHO Strategic Advisory Group of Experts (SAGE) revised their HPVV schedule recommendation, enabling countries to decide between a single-dose or two-dose schedule for non-immunocompromised girls aged 9–14 years. A recently published review of 55 studies (including clinical trials) across LMICs showed comparable efficacy and effectiveness between single-dose and multidose HPVV schedules in preventing persistent infection with HPV serotypes 16 and 18, which are responsible for most HPV-related cervical cancer cases globally, for up to 10 years postvaccination.^4^

At present, more than 45 countries have reported switching to a single-dose HPVV schedule or endorsed intent to adopt the single-dose HPVV recommendation.^5 6^ A single-dose regimen could yield significant programme benefits by reducing (in)direct costs of vaccine procurement/administration and improving global vaccine delivery through more equitable access. While the WHO and SAGE provide public health policy recommendations, they are not regulatory authorities; thus, at the time of their single-dose HPVV recommendation, this schedule remained ‘off-label’ in many countries, as existing vaccine licences from national regulatory bodies exclusively covered two-dose or three-dose HPVV.^47^,^13^ However, given the relative novelty of the permissive single-dose HPVV recommendation, there is limited understanding of country-level processes and key considerations informing the decision to adopt or reject a single-dose immunisation schedule.

Accordingly, we conducted a qualitative study across 19 LMICs in Africa and Asia—inclusive of existing HPVV programmes and forthcoming HPVV introductions, respectively—to characterise the decision-making landscape and multilevel drivers of (non-)adoption of the revised single-dose HPVV schedule recommendation. By uncovering the forces shaping early, delayed or non-adoption of the single-dose HPVV schedule, our findings can advance knowledge of factors enabling or disincentivising single-dose HPVV schedule implementation—supporting evidence-informed, country-led decision-making related to HPVV schedules in LMICs with established HPVV programmes or planned HPVV introductions.

## Methods

### Design and procedures

To identify countries from which relevant immunisation stakeholders would be purposefully sampled, we identified four discrete archetypes characterising LMIC progress along the HPVV introduction and single-dose adoption continua (see table 1): (1) Type I countries are newly introducing HPVV on a single-dose schedule; (2) Type II countries are planning to introduce HPVV but have not yet rendered a decision on the immunisation schedule or have chosen to introduce on a two-dose schedule; (3) Type III countries have already introduced HPVV on a two-dose schedule (ie, prior to the updated SAGE recommendations in 2022) and have switched to a single-dose HPVV schedule and (4) Type IV countries have already introduced HPVV on a two-dose schedule (ie, prior to the updated SAGE recommendations in 2022) but have not yet rendered a decision. On the immunisation schedule or have opted to continue with a multidose schedule.

**Table 1:** Country selection, by HPV vaccination (HPVV) introduction status and cervical cancer burden

Sampling typology	Country	Year of HPVV introduction	Cervical cancer burden estimates (per 100 000 population)	
			Incidence (cases)	Mortality (deaths)
Typology I: New HPVV introduction and adopted single-dose schedule	Bangladesh	2023	11.3	7.0
	Cambodia	2023	15.2	8.1
	India	2023	17.7	11.2
	Nigeria	2023	26.2	14.3
Typology II: New HPVV introduction and undecided on single-dose schedule	Ghana	Pending	27.0	16.9
	Madagascar	Pending	41.8	30.0
	Nepal	Pending	14.2	8.7
	Pakistan	Pending	5.4	3.6
Type III: Mature HPVV programme and adopted single-dose schedule	Burkina Faso	2022	15.9	13.0
	Cameroon	2020	33.1	25.7
	Côte d’Ivoire	2019	32.0	20.4
	Ethiopia	2018	22.3	16.8
	Laos	2020	12.0	6.5
	Tanzania	2018	64.8	42.2
Type IV: Mature HPVV programme and undecided on single-dose schedule	Eswatini	2023	95.9	64.3
	Indonesia	2016	23.3	13.2
	Kenya	2019	32.8	21.4
	Philippines	2016	15.5	8.0
	Uganda	2015	53.8	40.6

New introductions are defined as countries that introduced (or are planning to introduce) HPVV following the publication of the updated SAGE recommendations and WHO position paper on single-dose HPV vaccination (December 2022).

HPV, human papillomavirus; HPVV, HPV vaccination; SAGE, Strategic Advisory Group of Experts.

**Figure 1:**
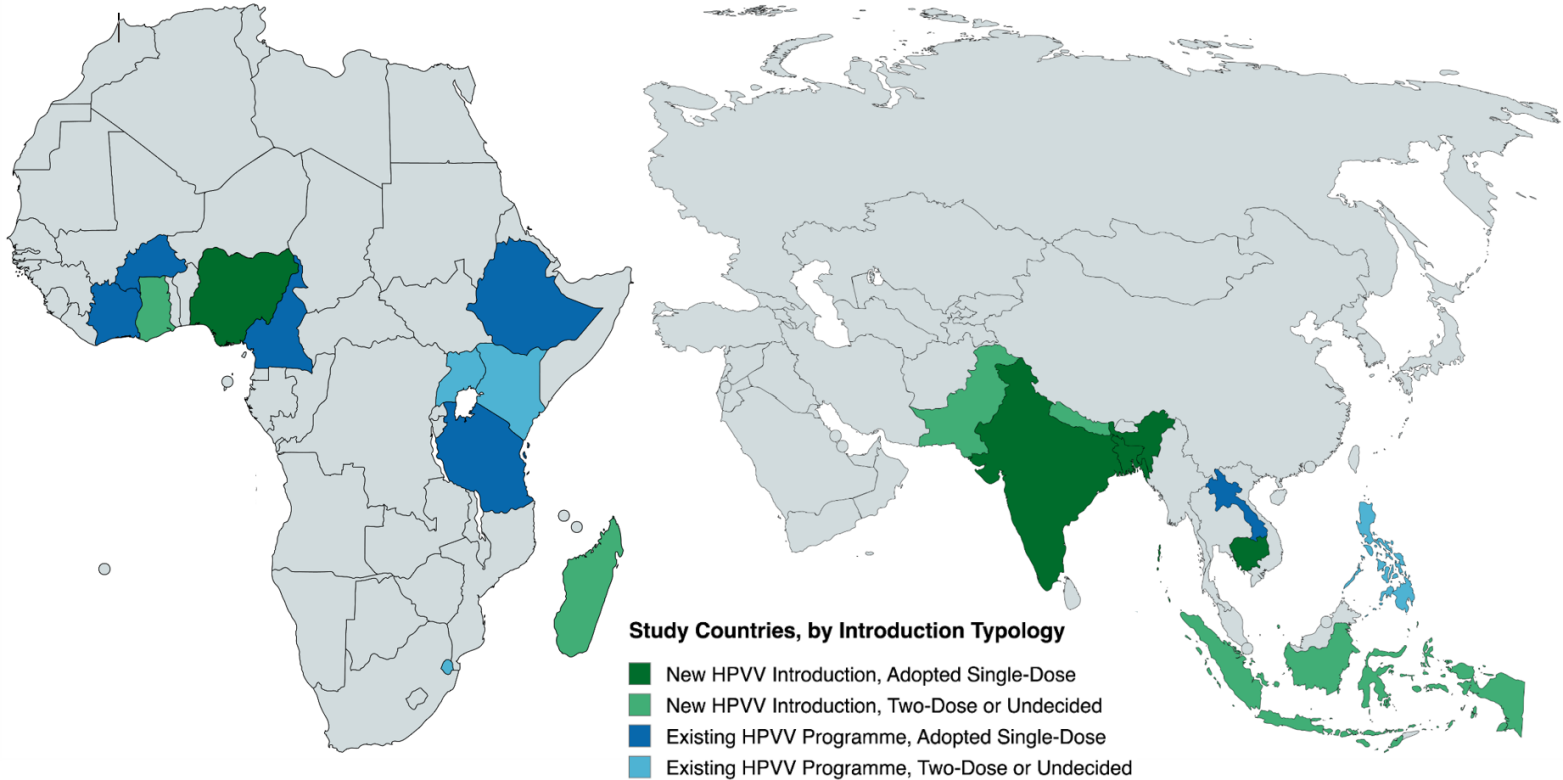
Countries sampled for study inclusion, by timing of HPV vaccination (HPVV) introduction and immunisation schedule. HPV, human papillomavirus.

Beyond the four identified country archetypes, our sampling strategy accounted for various country attributes that could influence single-dose decision-making processes and outcomes. Seeking variation in country characteristics (eg, HPVV programme maturity, planned or existing HPVV schedule, geographic region, eligibility for Gavi co-financing, cervical cancer burden), we employed a purposive, stratified sampling strategy,^14^ selecting countries based on the timing of their HPVV introductions and the outcomes of their national HPVV single-dose deliberations (see figure 1). This sampling approach would enable thematic comparisons across country archetypes and other emergent categories of interest (eg, region).

To elicit heterogeneous perspectives on country-level HPVV schedule-related decision-making, we identified relevant national immunisation stakeholders—who were sampled purposefully based on their professional affiliation—in consultation with Gavi and its core/expanded technical assistance partners. We identified additional individuals to approach via email for potential participation using snowball sampling (ie, through nomination and referral from interviewed key informants). To achieve thematic saturation, we aimed to recruit national immunisation stakeholders from a minimum of three countries per archetype. We also sought to conduct a minimum of three interviews per country for consistency and confirmability of emerging insights.^15^

From September 2023 to February 2024, we conducted virtual, semi-structured, key informant interviews in English and French with national immunisation stakeholders affiliated with Ministries of Health (MoH), Expanded Programmes for Immunisation (EPI), National Immunisation Technical Advisory Groups (NITAG), core/expanded HPVV technical assistance partners, civil society organisations and multilateral agencies across 19 African and Asian LMICs. Trained graduate-level qualitative data collectors (ER, IS, MDW, SR, CG) conducted 30–60 min interviews with key informants aided by a semistructured guide, allowing the conversation to proceed iteratively in pursuit of emerging themes. The semistructured guide covered the following topics: the status of national cancer control programmes, including HPVV services; factors and forces influencing HPVV schedule-related decision-making; stakeholders and actors involved in HPVV schedule-related decision-making; and processes and pathways to rendering decisions on the national HPVV schedule. Interviews were audio-recorded and professionally transcribed for subsequent analyses.

### Analysis

We used multicycle, iterative thematic analysis^16^ to characterise salient forces motivating (‘pull’ factors) or disincentivising (‘push’ factors) adoption of the HPVV single-dose schedule across the 19 LMICs included in the present study. First, we began by identifying overarching thematic domains and nested sub-constructs to catalogue core decision-making factors and processes surrounding HPVV schedule-related decision-making. Our initial approach to construct selection was guided by a modified version of Burchett *et al*’s New Vaccine Introduction Framework,^17^ which articulates sources of influence—operating at various socio-ecological levels—over new vaccine introductions, including HPVV, in LMICs.

After finalising a codebook in which thematic constructs were mapped to domains from the New Vaccine Introduction Framework (see table 2), we imported interview transcripts into ATLAS.ti V.9.0 (Scientific Software Development GmbH, Berlin, Germany) to facilitate data management and coding. Four coders (ER, IS, MDW, SR) working independently then coded each transcript once until codes had been applied to text segments across transcripts. To further synthesise emerging themes and identify conceptual patterns within the data, we exported coded text segments into Microsoft Excel and manually reassembled themes with the aid of a hierarchical array,^18^ which facilitated reorganisation of coded text segments by various country attributes (see online supplemental file 1). Finally, analytic memo-writing, weekly debriefs among the investigative team and concurrent dissemination of emergent findings to global immunisation stakeholders enabled continuous refinement and crystallisation of salient themes and data patterns.

**Table 2:** Identified codes derived from the New Vaccine Introduction Framework, by thematic domain

Thematic domain	Subdomain (Code)	Definition
Population health and political considerations	NITAG formation	Presence and formation of a functional NITAG (or lack thereof) influenced SDDM timing
	EPI functionality	Robust EPI (or lack thereof) guided SDDM
	Presence of high-risk/key groups	Presence (or absence) of high-risk groups (eg, OOS girls, girls/women living with HIV) shaped SDDM
	Cervical cancer burden/interventions	Cervical cancer incidence, mortality and complementary interventions (or lack thereof) guided SDDM
	Vaccine coverage and equity opportunities	Considerations related to HPV vaccine coverage and expansion to unreached populations guided SDDM
	Early adopters	Evidence of other countries adopting the single-dose schedule (or lack thereof) shaped SDDM
	Public health and political priorities	Politics and competing public health priorities (eg, armed conflict, disease outbreak) influenced SDDM
	Vaccine hesitancy/acceptance	Anticipated acceptance or reticence towards a new vaccine schedule influenced SDDM
Vaccine characteristics	Single-dose evidence	Existing immunogenicity and efficacy evidence of single-dose (or lack thereof) guided SDDM
	Schedule performance	Considerations related to vaccine schedule (eg, complexity of dose timing) guided SDDM
	Off-label use	Implications of single-dose off-label use and experience with other off-label products shaped SDDM
Programme considerations	Service integration/routinisation potential	Ability (or lack thereof) to integrate HPV vaccines into other services or routine delivery guided SDDM
	Adaptation logistics	Requirements (or lack thereof) to adapt existing training materials, pamphlets, or job aids influenced SDDM
	Storage/stock management capacity	(In)ability to store and maintain excess product shaped SDDM
	Programme timelines	Deadlines/timelines related to programme planning (eg, delayed MACs, campaigns) influenced SDDM
	Delivery models	Planned or existing vaccine implementation models (eg, school-based, facility-based) shaped SDDM
	Supply chain	Vaccine product availability or constraints shaped SDDM
Financial/economic considerations	Costs/cost-effectiveness and sustainability	Anticipated/realised costs associated with the national HPV vaccination programme informed SDDM
	Funding sources	Payment sources (eg, Gavi, pharmaceuticals, philanthropies) for products/programmes influenced SDDM
	Vaccine price	Manufacturing, procurement and distribution costs of vaccine products guided SDDM
Sources of influence	Pharmaceutical industry	Lobbying/advocacy by pharmaceutical companies and vaccine manufacturers influenced SDDM process
	NITAG/RITAG	Immunisation technical working groups (national or regional) influenced SDDM process
	SAGE recommendation	SAGE recommendation for single-dose HPV vaccination influenced SDDM process
	WHO position paper	WHO position paper endorsing single-dose HPV vaccination influenced SDDM process
	Gavi and donors	Gavi and other donor organisations (eg, Bill & Melinda Gates Foundation) influenced SDDM process
	Country line ministries	Country ministries (eg, Health, Education, Finance) influenced SDDM process
	Professional associations/societies	Professional groups (eg, advocacy groups, physician/nursing associations) influenced SDDM process
	Academia/research	Researchers, scientists and/or academics influenced SDDM process

EPI, Expanded Programme on Immunisation; HPV, human papillomavirus; MAC, Multi-age cohort; NITAG, National Immunisation Technical Advisory Group; OOS, out-of-school; RITAG, Regional Immunisation Technical Advisory Group; SAGE, Strategic Advisory Group of Experts on Immunisation; SDDM, single dose decision-making.

### Reflexivity

Our investigative team consisted primarily of cisgender women and individuals with minoritised racial and ethnic identities, including three foreign-born researchers from LMICs. Four of the researchers had over a decade of experience with research and/or programming related to immunisations, sexual and reproductive health and/or maternal and child health in LMICs. The products of this research emanate from collaborative, reciprocal partnerships between the investigative team, based at a research institute in a high-income country, and HPVV implementers/decision-makers globally, many of whom are based in the LMICs included in the present study.

### Patient and public involvement

Patients and members of the public were not involved in the design, implementation or interpretation of findings emanating from the present study.

## Results

Between September 2023 and February 2024, we contacted 167 national immunisation stakeholders, of whom 99 (59.3%) responded to express interest in participating in the study. Stakeholders from four countries—Benin, Guyana, Solomon Islands and Tunisia—did not respond to recruitment-related communications. Of these, 66 stakeholders (66.7%) across 19 African and Asian LMICs enrolled in the study and completed a key informant interview (see table 3). The largest fraction of stakeholders included MoH/EPI affiliates (n=26, 39.4%), followed by core/expanded technical assistance partners or civil society organisations (n=22, 33.3%) and multilateral agencies (n=18, 27.3%).

**Table 3:** Descriptive characteristics of immunisation stakeholders from 19 low-income and middle-income countries completing key informant interviews (N=66)

Characteristics	Number (n)	Per cent (%)
Region		
Central and South Asia	12	18.2
East and Southern Africa	20	30.3
Southeast Asia	13	19.7
West and Central Africa	21	31.8
HPVV introduction typology		
Type I: new introduction, adopted single-dose	11	16.7
Type II: new introduction, two-dose or undecided	15	22.7
Type III: existing programme, adopted single-dose	26	39.4
Type IV: existing programme, two-dose or undecided	14	21.2
Affiliation		
Ministry of Health/EPI	26	39.4
Implementing partner/CSO	22	33.3
Multilateral agency	18	27.3

CSO, civil society organisation; EPI, Expanded Programme on Immunisation; HPVV, human papillomavirus vaccination.

### ‘Pull’ factors: perceived advantages and benefits of single-dose HPVV

Stakeholders across all LMICs described key advantages and perceived benefits of a single-dose HPVV schedule, specifically evidence-based support (ie, vaccine efficacy data, endorsements by global bodies, influence of other countries adopting a single-dose HPVV regimen); anticipated improvements to population health (ie, reduced cervical cancer burdens, increased vaccination coverage); and perceived logistical and financial advantages (ie, costs savings, streamlined programme operations, optimised stock management) (see online supplemental file 1).

#### Vaccine efficacy evidence

The decision by LMICs to adopt the single-dose schedule for HPVV was primarily driven by the presence of robust vaccine efficacy data. Stakeholders across countries consistently pointed to the robust evidence base, which established the non-inferiority of single-dose HPVV relative to a two-dose regimen, affirmed the safety and immunogenicity of mono-dose HPVV. These data empowered national immunisation decision-makers to entertain single-dose HPVV as an alternative to well-established multidose schedules.

‘Here are the studies. The evidence is clear, and single-dose [HPVV] has comparable efficacy to two doses for girls in the general population. And we must not hesitate. We must advance.’ –Côte d’Ivoire (Existing HPVV Programme, Switched to Single-Dose Schedule)

#### Endorsement by global scientific bodies

Many stakeholders framed the SAGE recommendation and WHO position paper as the forces initially triggering discussions about revisiting the HPVV schedule, bolstering country confidence in the safety, efficacy and programme benefits of single-dose HPVV. Endorsements from these prolific global entities were especially important, given that a single-dose schedule would likely entail off-label use of licensed HPVV products. Some stakeholders even explained how the SAGE recommendation and WHO position paper empowered decision-makers to counter single-dose HPVV opposition by vaccine manufacturers, who advocated fervently for multidose schedules. Importantly, while stakeholders considered the SAGE and WHO recommendations ‘gold standards’, they simultaneously emphasised deliberate processes undertaken to contextualise the single-dose HPVV recommendation within their respective country’s epidemiologic, fiscal and sociopolitical environments.

‘What is the role of the WHO? It is really to ensure that the Minister’s decision-making is made in an informed manner, taking into account all the directives—in particular, the WHO directives—that exist.’ –Cameroon (Existing HPVV Programme, Switched to Single-Dose Schedule)

#### Decision-making and implementation precedents in LMICs

The availability of documented experiences and lessons learnt from countries that had already adopted and implemented HPVV on a single-dose schedule, including high-income countries, proved to be a valuable resource for decision-makers. Stakeholders universally cited this as a factor that motivated their adoption of the single-dose schedule. Another recurring theme was the strong desire for more contextualised evidence from other LMICs. Stakeholders highlighted the importance of information-sharing among LMICs, especially those within their region, and advocated for additional research, evaluations and documentation of lessons learnt from countries implementing a single-dose schedule. For stakeholders from African countries in particular, these relatable experiences served as valuable references and fostered confidence in the feasibility and effectiveness of single-dose HPVV implementation in similar contexts.

‘We requested [results from] other countries that had switched to single-dose [HPVV] or are looking to switch to single-dose [HPVV], particularly countries in Africa that had a similar context to Nigeria and are using the same product. We typically learn lessons and gather information from other countries in Africa to inform our decision.’ –Nigeria (New HPVV Introduction, Adopted Single-Dose Schedule)

‘For middle-income countries, they look at other countries, either which are higher-income or equivalent…For example, if a country sees that Australia or the United Kingdom has gone to a single dose, it becomes easier than if you tell them that South Africa or Kenya or Moldova has gone to a single dose.’ –Philippines (Existing HPVV Programme, Undecided on Single-Dose Schedule)

#### Improved programme performance and vaccination coverage

The potential for a single-dose schedule to increase population-level protection against cervical cancer was a key motivator of single-dose HPVV adoption in many countries. Stakeholders described the many difficulties associated with second-dose HPVV administration and were enthusiastic about reducing attrition between the first and second doses.

‘The two-dose [or] three-dose schedule of HPV [vaccination] is really difficult programmatically, and we are experiencing high dropout [rates]. We are actually missing vulnerable and at-risk girls. And implementing it with two doses [or] three doses has reinforced inequities…Those are considerable issues.’ –Ethiopia (Existing HPVV Programme, Switched to Single-Dose Schedule)

Stakeholders also anticipated improved vaccination coverage and completion rates, particularly among harder-to-reach populations like out-of-school girls, nomadic communities, zero-dose populations and marginalised ethnic minorities. Across regions, countries that adopted the single-dose HPVV schedule were motivated by the presence of under-immunised and high-risk groups (ie, immunocompromised girls including those living with HIV or sickle cell anaemia). While these underimmunised groups represented only a small fraction of a country’s population, the prospect of improving vaccination coverage in these strata buttressed decision-makers’ optimism towards single-dose HPVV.

*‘*Zero-dose [populations] are one of the major concerns because even in the routine immunization [programme], there are about 1 million children every year who are never reached with vaccines. It is known that there could be some zero-dose populations [to consider in HPVV planning].’ –Ghana (New HPVV Introduction, Undecided on Single-Dose Schedule)

In addition to addressing immunisation coverage disparities and promising more equitable protection across population strata, the single-dose schedule was also framed as an opportunity for countries with existing HPVV programmes to expand their programme scope to include gender-neutral and expanded age-cohort vaccination strategies. Stakeholders who advocated for gender-neutral HPVV strategies explained that it was not only a more efficient way to interrupt HPV transmission, but also necessary to mitigate vaccine hesitancy (often fuelled by rumours of vaccine-induced infertility), which was a persistent barrier to HPVV uptake in some contexts.

*‘*Shifting from two to one dose is an opportunity to extend the vaccination to boys, knowing the risk of HPV amongst boys…And this is aligned with cultural norms among many people in Cameroon, who are not comfortable with something targeting only girls. If it is only targeting boys, it is more acceptable. But [if it is] only young girls at school, it’s really a big concern for the population.’ –Cameroon (Existing HPVV Programme, Switched to Single-Dose Schedule)

#### Reduced cervical cancer burdens

Immunisation stakeholders from countries with outsized cervical cancer burdens cited the promise of expanded population immunity vis-à-vis higher immunisation coverage as a principal driver of single-dose HPVV adoption. Because a single-dose schedule was perceived as a key antecedent to increased HPVV coverage, single-dose HPVV demonstrated great potential to reduce the economic and social costs of cervical cancer.

*‘*Nepal has a very high burden of cervical cancer. It’s the highest amongst the cancers for females in Nepal, so there’s a lot of mortality, morbidity and expenditure. This is a priority disease to be treated and prevented.’ –Nepal (New HPVV Introduction, Undecided on Single-Dose Schedule)

Some stakeholders also expressed optimism about the alignment of the single-dose schedule with global efforts to achieve the 90-70-90 targets for cervical cancer elimination,^19^ framing HPVV as ‘low-hanging fruit’ for prevention efforts, especially when access to cervical cancer screening and treatment remains low.

*‘*One of the factors was we have maximum political acceptability towards the vaccine. Why is this? Because the cervical cancer is still among the leading cancer in our country, and the only intervention which is in place to prevent that is vaccination. So politically, it draws maximum attention.’ –Tanzania (Existing HPVV Programme, Switched to Single-Dose Schedule)

#### Lower vaccine procurement costs

While not all countries discussed vaccine price, stakeholders who did emphasise the potential for relative cost savings due to lower vaccine procurement demands, with a single dose per person translating directly to decreased programme expenses.

*‘*Kenya is expected to be self-financing by 2030, so then the Government is supposed to take 100% of the cost of vaccines in Kenya. Therefore, a less costly vaccine or a less costly schedule is welcome if the evidence shows that it is just as efficacious, just as effective, and just as safe.’ –Kenya (Existing HPVV Programme, Undecided on Single-Dose Schedule)

#### Circumventing vaccine supply constraints

Given the ongoing global shortage of HPVV products (which have historically required multidose regimens), some stakeholders perceived the single-dose approach as a potential solution to alleviate demand pressures on a limited global vaccine supply. They explained that a single-dose schedule drastically reduces the total number of doses required to vaccinate a target population, lowering the overall procurement costs per vaccinated individual. Such cost efficiencies were especially attractive for countries supported by Gavi for programme implementation and forecasted to transition out of Gavi support in the near future, enabling them to maximise the reach of available vaccine supply within budgetary constraints.

*‘*They are still concerned about the vaccine availability because there still appears to be global supply challenges. Because of that occasional on-and-off supply challenge, they are a little bit concerned about the possibility of having this situation in the future. Or, because now we have a single-dose [HPVV schedule], does it mean that technically there will be more vaccines available than originally [planned]?’ –Ghana (New HPVV Introduction, Undecided on Single-Dose Schedule)

#### Streamlined programme operations

While single-dose adoption would not change service delivery strategies (ie, primarily school-based or community-based and facility-based for out-of-school girls), stakeholders further explained that shifting to a single-dose schedule would eliminate the need for activities associated with a two-dose regimen, such as defaulter tracing and maintaining longitudinal dose registers. Furthermore, HPVV falls outside the purview of childhood vaccination programmes, oftentimes requiring overworked and under-resourced healthcare workers to collaborate with schools for vaccine delivery. Single-dose administration would, thus, translate to potential cost savings by reducing operational resources required for programme implementation.

*‘*Planning for the second dose [of HPVV] is quite difficult, and you have to use a lot of resources. [HPVV] is not usually included in the immunisation programme. We were used to vaccinating children under five and, most of the time, children under two years. You have extended the margin of the [health care workers’] responsibilities…Now, the healthcare workers must go to the schools, plan with the schools, and do the registration.’ –Tanzania (Existing HPVV Programme, Switched to Single-Dose Schedule)

While only one stakeholder from Uganda, Bangladesh and Indonesia each indicated their NITAGs considered specific, cost-effectiveness evidence (from peer-reviewed literature or country-level research conducted by international technical assistance partners), nearly all countries’ stakeholders discussed reviewing their national budgets and anticipated cost savings as a key benefit of adopting a single-dose schedule.

#### Optimised storage and stock management

While not all countries discussed stock management and capacity, those who did emphasised how adopting a single-dose HPVV schedule would streamline operations associated with storage and stock management at healthcare facilities—an important consideration for low-income countries, especially those in Africa managing multiple vaccine introductions simultaneously. Stakeholders reported that recent COVID-19 vaccination efforts led to improved stock management in many LMICs, due to substantial investments in cold chain capacity (often with support from international organisations to facilitate vaccine rollout) and the application of lessons learnt about digital inventory and management systems to HPVV. Despite these improvements, which enabled countries to manage higher vaccine volumes, stakeholders asserted that the single-dose approach still provided a significant advantage. Most stakeholders were, however, careful to clarify that while these logistic advantages were appreciated, they were neither sufficient nor necessary conditions for adopting the single-dose schedule.

*‘*Of course, the cold chain is a big factor, but now after [the COVID-19] pandemic…we have enough cold chain capacity because we have received [funds], and we have extended our cold chain buildings to accommodate more vaccines. At present, there is no cold chain problem at the primary, secondary, or tertiary level. There is enough space [for HPVV].’ –Bangladesh (New HPVV Introduction, Adopted Single-Dose Schedule)

### ‘Push’ factors: anticipated challenges and perceived disadvantages of single-dose HPVV

Stakeholders identified several factors as demotivating HPVV single-dose schedule adoption or delaying decision-making on the HPV immunisation dosing schedule. In this section, we describe salient factors that deterred countries from adopting the single-dose HPVV schedule, including stakeholder concerns about existing single-dose efficacy evidence, specifically limited data on long-term protection, and the off-label use of the single-dose approach. Anticipated logistical challenges with single-dose schedule implementation and perceived financial implications of single-dose HPVV also emerged as barriers to single-dose adoption. Specifically, these anticipated challenges/barriers included concerns related to effectively communicating a rationale for the switch from two- to one-dose HPVV, costs/resources associated with adapting training and communication materials, and inadvertent widening of inequities in HPVV coverage (see online supplemental file 1).

#### Limited localised evidence on durability of protection

Countries consistently expressed concerns about the duration of protection from a single-dose schedule and the limited published evidence of long-term immunogenicity, especially in Asian and African contexts, as well as the absence of any data on clinical endpoints (ie, cervical cancer). The lack of long-term data on immune response following single-dose HPVV raised questions about the durability of mono-dose HPVV’s protection. To allay concerns about limited localised evidence of long-term immunologic protection from single-dose HPVV, some countries planned to conduct additional research through their national surveillance systems to evaluate long-term immunogenicity within their own populations. Stakeholders expressed their desire for more localised evidence on prevalent HPV serotypes to ensure their selected vaccine products were appropriate for their epidemiologic contexts.

*‘*Obviously, the off-label use also triggered discussions. It’s like the manufacturers are not bearing any responsibility if the vaccine is not producing immunity. This is also why the NITAG strongly recommended that there be a surveillance system that can capture immunogenicity among girls to ensure they are being protected. It didn’t trigger a “No” response, but it triggered the sentiment that we need to establish the adequate structure to monitor vaccine effectiveness.’ –Burkina Faso (Existing HPVV Programme, Switched to Single-Dose Schedule)

#### Off-label use concerns

Numerous countries had prior experience with off-label use of other immunisation products, and while off-label use alone did not tend to discourage single-dose HPVV adoption, it potentiated existing concerns about evidence gaps. Stakeholders in some countries worried about liability if adverse events occurred in the context of off-label HPVV product use. Off-label use concerns were also more salient in countries with larger pockets of vaccine-hesitant populations, where clear communication regarding the off-label use of HPVV products was anticipated to be a significant challenge. Stakeholders further expressed concern that healthcare workers and the public might be more apprehensive about a new vaccination schedule deviating from well-established immunisation guidelines, even when clear precedents for off-label vaccine administration (eg, Polio, Pneumococcal) existed. In Indonesia, for example, the NITAG and MoH were reticent to endorse a single-dose HPVV schedule in the absence of immunisation products licensed for single-dose implementation.

‘You can be worried about the efficacy. That part probably makes sense. But when they started talking about the fears of vaccine hesitancy due to the off-label strategy, then it started to lock us.’ –Indonesia (Existing HPVV Programme, Undecided on Single-Dose Schedule)

Separately, in the Philippines, negative experiences with the Dengvaxia tetravalent dengue vaccine fostered reticence towards off-label use of vaccine products. Consequently, even the WHO position paper recommending single-dose HPVV was insufficiently persuasive in HPVV schedule-related deliberations.

*‘*If the pharmaceutical [manufacturers] would have it accredited as a single-dose [product] with the Food and Drug Administration, then it would just be a breeze for the country. We’re seeing that the Department of Health is leaning towards single-dose [HPVV], but there’s the issue of technical legalities because of the Dengvaxia experience.’ –Philippines (Existing HPVV Programme, Undecided on Single-Dose Schedule)

#### Challenges communicating a rationale for single-dose HPVV

A primary concern for countries with mature HPVV programmes was effectively communicating the rationale behind the switch from a multidose to a single-dose schedule to both healthcare workers and the public. Some stakeholders worried about the potential for rumours to circulate in which the schedule switch would be portrayed as a cost-cutting measure. Stakeholders noted that clear messaging highlighting the scientific rationale for single-dose HPVV was essential to maintaining public trust and strengthening health workforce and community confidence in a new immunisation schedule.

‘I think the biggest concern, in terms of implementation, is how to avoid confusion amongst community members. And not only to avoid confusion, but also to manage the confusion or to come up with the standard operating procedures for the healthcare provider as well.’ –Laos (Existing HPVV Programme, Switched to Single-Dose Schedule)

Stakeholders from Cameroon, which had low two-dose HPVV coverage, explained that developing relatable and trustworthy health communications required immunisation programmes to anticipate specific questions, like whether the two-dose regimen was an ‘overdose’. Some stakeholders explained that working with community representatives and civil society organisations, even as early as when NITAG deliberations on the immunisation schedule began, helped them to anticipate and address these concerns. Interestingly, stakeholders from Cameroon also perceived their adoption of a gender-neutral strategy along with the single-dose approach as an opportunity to allay concerns about the schedule switch, especially in the context of vaccine hesitancy.

*‘*The switch from two-dose to single-dose [HPVV] and from girls [only] to gender-neutral [vaccination] was very helpful to shape and adapt communication strategies to raise the risk perception among the targeted population.’ –Cameroon (Existing HPVV Programme, Switched to Single-Dose Schedule)

#### Widening HPVV coverage inequities

Stakeholders also expressed concerns about the potential for a single-dose schedule to widen HPVV coverage inequities, especially in countries with higher HIV burdens—where girls and women living with HIV would still require multiple doses for adequate protection. The adoption of a single-dose schedule could further marginalise these populations and exacerbate disparities in access to cervical cancer prevention and treatment services, undermining efforts to reduce morbidity and mortality in disproportionately impacted populations. Some countries also considered the operational challenge of ensuring persons living with HIV would continue to receive the appropriate vaccine dosage, without inadvertently disclosing girls’ HIV status.

*‘*I think that the other thing that was considered heavily was those girls living with HIV, who will continue to receive a second dose [of HPVV]. There were in-depth discussions about how we discreetly reach these girls to deliver their second dose, so we don't ‘out’ their HIV status.’ –Eswatini (Existing HPVV Programme, Undecided on Single-Dose, Schedule)

## Discussion

LMIC decisions to adopt or switch to a single-dose HPVV schedule reflect a dynamic interplay between scientific evidence, programme costs and (dis)advantages, and the potential for significant public health impact. Robust scientific evidence and endorsements from WHO and SAGE were oftentimes sufficient for LMICs to proceed with HPVV single-dose deliberations and adoption, even amidst off-label use and other programme/financial concerns. Country-level experiences from early adopters, including high-income countries (eg, Australia), further bolstered confidence in single-dose HPVV. As seen with fractional-dose inactivated polio vaccine (fIPV),^20^ rapid dissemination of localised implementation evidence should be prioritised to build confidence in the safety, effectiveness and operational feasibility of the single-dose HPVV schedule.

A key driver of single-dose HPVV schedule adoption across countries was the potential for the single-dose approach to significantly improve vaccine coverage and reduce cervical cancer burdens, particularly among harder-to-reach, underserved and disproportionately impacted populations. Some LMICs with existing vaccine supply also viewed single-dose as an entry point for expanding vaccination to other age groups or implementing gender-neutral vaccination, which has been described as an effective approach for halting HPV transmission at the population level.^21 22^ However, in some countries (notably those with elevated HIV burdens), there is concern that a single-dose strategy may widen underlying HPVV coverage inequities in specific populations (ie, girls living with HIV) who require additional vaccine doses for adequate protection. Countries adopting single-dose HPVV must ensure that populations for whom one dose of HPVV is insufficiently protective are provided convenient, private, affordable and accessible alternatives for accessing supplemental vaccine doses^23 24^—potentially in venues where existing touchpoints for health service provision to these populations already exist (eg, HIV clinics, retail pharmacies).

Cost savings were attractive for resource-limited settings, and stakeholders emphasised that improved programme efficiency and the potential to alleviate pressure on limited vaccine supplies were significant advantages to single-dose HPVV. While stakeholders framed the decision to adopt a single-dose schedule as primarily driven by the promise of improved coverage, the two are inextricably linked: if countries cannot afford to vaccinate their populations, then their coverage will plummet. While potential cost savings associated with single-dose HPVV are understandably attractive, especially to LMICs, there is a risk that these savings may not be reinvested within HPVV programmes, as the MoH/EPI may instead choose to reallocate these funds to strengthen other immunisation activities, inadvertently weakening the HPVV programme infrastructure.^24 25^ To ensure maximum impact and sustainability, it is imperative that any costs saved from switching to a single-dose HPVV schedule are reinvested (rather than reallocated) within national HPVV programmes.

Uncertainties regarding off-label use and long-term immunogenicity from single-dose HPVV emerged as salient barriers to schedule adoption for some countries, highlighting the need for both continued evidence generation focused on long-term protection in diverse populations and effective communication strategies to allay these concerns and bolster public confidence in the single-dose schedule. Ongoing monitoring and evaluation efforts will be essential to assess the long-term effectiveness and impact of the single-dose HPVV implementation in LMICs. Prior harms attributed to off-label use of vaccine products, like those described in some Southeast Asian countries, demonstrate how negative experiences can diminish enthusiasm for a programme-optimising single-dose schedule, even in the presence of robust safety data and endorsement from authoritative health bodies.^26 27^ Addressing these concerns and building trust in the single-dose approach will be crucial for wider adoption globally. Countries considering single-dose adoption can support decision-makers by drawing parallels to safe and effective off-label use of other immunisation products, like fIPV.^20^ This may also require communications that effectively decouple implementation of novel immunisation products like Dengvaxia from novel off-label use of existing vaccine products like HPVV.

In summary, this study offers several key insights for improving the adoption and implementation of single-dose HPVV:

- **Leverage existing precedents**: Communicating the successful off-label application of other immunisation products can help overcome hesitancy towards single-dose HPVV.
- **Ensure timely evidence dissemination**: Rapidly synthesising and sharing new single-dose evidence with national immunisation stakeholders and the immunisation workforce can build confidence in single-dose HPVV.
- **Develop tailored communication strategies**: Effectively addressing misconceptions about single-dose HPVV requires context-specific communication strategies that engage diverse stakeholders across various sectors and civil society.
- **Facilitate knowledge sharing**: Sharing implementation experiences and lessons learnt from single-dose HPVV implementation, particularly among neighbouring LMICs, can foster greater confidence in adopting a single-dose schedule.
- **Strategically reinvest cost savings**: Cost savings realised by transitioning from a two-dose to a single-dose HPVV schedule should be reinvested within national HPVV programmes to maximise their reach and impact.

### Limitations

Study findings are subject to several limitations. First, interviews were conducted cross-sectionally and, thus, may not capture the evolving landscape of drivers of HPVV schedule-related decision-making. Second, despite our best efforts to include a diverse body of countries and stakeholders in the study, there may be inherent differences between stakeholders who opted to participate and those who were unavailable, unresponsive or declined to participate. Third, insights obtained from interviewed stakeholders may have limited transferability to stakeholders and countries not represented in the study. Finally, stakeholders may have self-censored and avoided disclosing potentially sensitive information about single-dose decision-making, especially in contexts where a decision on the HPVV schedule was still pending at the time of data collection.

## Conclusions

Our qualitative study across 19 African and Asian LMICs highlights the dynamic decision-making processes surrounding the adoption of the updated single-dose HPVV schedule recommendation, highlighting both enabling and constraining forces at country level. While HPVV programmes are in an era of revitalisation (following a period of dormancy during COVID-19), and cervical cancer elimination remains a global health priority, future vaccine introductions by LMICs should evaluate if the existing framework and structure in their country is conducive to agile vaccine policy decision-making.^28^ A functional NITAG is needed to guide a country towards immunisation schedule decisions, and future vaccine introductions by LMICs should prioritise this to facilitate HPVV schedule-related decision-making. Taken together, our study’s findings can support single-dose HPVV advocates as they work to expedite evidence-informed, country-led decision-making in an increasingly complex immunisation ecosystem.

## Supplementary material

10.1136/bmjgh-2024-018779 online supplemental file 1

## Data Availability

Data are available on reasonable request.
